# Smc5/6 complex regulates Sgs1 recombination functions

**DOI:** 10.1007/s00294-016-0648-5

**Published:** 2016-09-23

**Authors:** Marcelino Bermúdez-López, Luis Aragon

**Affiliations:** Cell Cycle Group, MRC Clinical Sciences Centre, Imperial College, London, W12 0NN UK

**Keywords:** Smc5/6 complex, Sgs1, Top3, SUMOylation, Homologous recombination, DNA damage

## Abstract

The family of RecQ helicases is evolutionary conserved from bacteria to humans and play key roles in genome stability. The budding yeast RecQ helicase Sgs1 has been implicated in several key processes during the repair of DNA damage by homologous recombination as part of the STR complex (Sgs1-Top3-Rmi1). Limited information on how is Sgs1 recruited and regulated at sites of damage is available. Recently, we and others have uncover a direct link between the Smc5/6 complex and Sgs1. Most roles of Sgs1 during recombination, including DNA end resection, Holiday junction dissolution, and crossover suppression, are regulated through Mms21-dependent SUMOylation. Smc5/6 first acts as a recruiting platform for STR and then SUMOylates STR components to regulate their function. Importantly, the assembly of STR is totally independent of Smc5/6. Here, we provide a brief overview of STR regulation by Smc5/6.

**Electronic supplementary material:**

The online version of this article (doi:10.1007/s00294-016-0648-5) contains supplementary material, which is available to authorized users.

## Introduction

DNA damage, originates from endogenous and exogenous sources, and is constantly challenging genome integrity. To overcome this threat, cells have evolved a complex network of intertwined DNA damage repair pathways. Failures in these lifeguard systems compromise genome integrity, which can lead to genome instability, cancer predisposition, and ageing. The RecQ helicases are highly conserved family of proteins from bacteria to humans with critical roles in the maintenance of genome stability over generations. Humans have five distinct RecQ members: BLM, WRN, RECQL1, RECQL4, and RECQL5, whereas only two RecQ-like helicases have been described in budding yeast, Sgs1, and Hrq1 [reviewed in (Bernstein et al. 2010)].

Mutations in BLM cause Bloom syndrome (BS), a genetic disorder characterized by a strong growth deficiency, reduced fertility, diabetes, and cancer predisposition (German et al. 2007). BS cells show increased frequencies of sister chromatid exchanges (SCE), quadriradial chromosome configurations (Chaganti et al. 1974; German et al. 1974), elevated mutation rates and defects in DNA replication expressed as an accumulation of aberrant intermediates (Lonn et al. 1990) and slower replication rates (Hand and German 1975). Over the past decades, many studies have been focused on understanding the functions of BLM during DNA repair. Since the phenotypes of *sgs1∆* cells resemble those observed in BS cells, including replication defects and hyper-recombination (Bernstein et al. 2009), many insights from yeast have been applicable to BLM. Both BLM and Sgs1 form a heteromeric complex with the type IA topoisomerase TOPIIIα (Top3 in yeast) (Bennett et al. 2000; Gangloff et al. 1994; Oakley et al. 2002), and the OB-fold RecQ-mediated instability factors, RMI1 and RMI2 (Rmi1 in yeast) (Chang et al. 2005; Mullen et al. 2005) and both complexes have been shown to play extensive roles during DNA repair.

## Functions of BLM/Sgs1 during homologous recombination

The BLM-TOPIIIα-RMI1-RMI2 (BTRR) complex in humans and the Sgs1-Top3-Rmi1 (STR) complex in budding yeast are key players in various processes of DNA metabolism, such as replication, DNA repair, and telomere maintenance. These functions have been extensively reviewed in several journals (Croteau et al. 2014; Manthei and Keck 2013)

Many studies have revealed that BLM/Sgs1 is a central element in the homologous recombination (HR) process, where it carries both pro- and anti-recombinogenic functions. First, BLM/Sgs1, together with DNA2 and EXO1, promotes DSB resection (Gravel et al. 2008; Nimonkar et al. 2011; Nimonkar et al. 2008). Second, it regulates D-loop formation depending on the ADP/ATP-bound state of RAD51. When RAD51 is in its inactive ADP-bound form, BLM can disrupt the RAD51-ssDNA filament preventing the D-loop formation (Bugreev et al. 2007). On the contrary, when RAD51 is in its active ATP-bound form, BLM promotes RAD51 homology search, strand invasion, and, therefore, D-loop formation (Bugreev et al. 2009). Moreover, several studies have shown that BLM/Sgs1 may have a role in synthesis-dependent strand annealing (SDSA) by disrupting D-loops (Bachrati et al. 2006; van Brabant et al. 2000). Third, BTR and STR complexes form the “dissolvasome” which is involved in the processing of late recombination intermediates during the separation of double Holliday junctions (dHJs). Importantly, during the dissolution of dHJs, the helicase activity of BLM/Sgs1 and the topoisomerase activity of TOPIIIα/Top3 are combined to yield products without associated crossovers (Bzymek et al. 2010; Cejka et al. 2010; Ira et al. 2003; Plank et al. 2006; Wu and Hickson 2003). Moreover, the complex promotes the resolution of ultra-fine bridges that can jeopardise chromosome segregation in mitosis (Chan et al. 2007).

BLM/Sgs1 also performs an important role during DNA replication by stabilising stalled forks (Cobb et al. 2003). BLM is present in the advancing replication fork (Alabert et al. 2014), and some results suggests that BLM interacts with FEN1 and thereby assists in the maturation of Okazaki fragments (Bartos et al. 2006; Sharma et al. 2004. Sgs1 is also important for the processing of recombination-dependent structures that appear at damaged forks (Liberi et al. 2005; Mankouri et al. 2007). Because of these roles, BS cells are hypersensitive to drugs that interfere with DNA replication (Davies et al. 2004), and show a higher percentage of fork stalling (Rao et al. 2007). BLM/Sgs1 is involved in replication fork restart after replicative stress (Davies et al. 2007), perhaps, by assisting fork regression (Machwe et al. 2011).

BS cells display telomere defects, such as sister chromatid linkages and telomere loss, which indicate that BLM has a role in processing late-replication intermediate structures that arise during telomere replication (Barefield and Karlseder 2012). It is also involved in the alternative lengthening of telomeres (ALT), a recombination-based mechanism for telomere maintenance. BLM localizes at telomeres specifically in ALT cells and interacts with proteins of the shelterin complex that modulates BLM and WRN unwinding activities on structures that mimic the T-loop in vitro (Lillard-Wetherell et al. 2004; Opresko et al. 2005; Opresko et al. 2002).

## Regulation of STR by the Smc5/6 complex

**Fig. 1:**
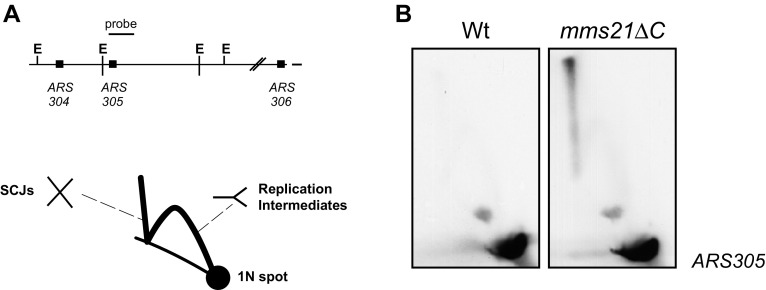
*mms21∆C* cells accumulate HJs at damaged forks. *Left panel* genomic region containing *ARS305* origin of replication and probe used for hybridization. Schematic representation of structures visualized by 2D gel electrophoresis. *Right panel* 2D gel electrophoresis of wild-type and *mms21∆C* cells. Cells were arrested in G1 with α factor. Once arrested, cells were released from G1 arrest into fresh medium containing 0.033 % MMS for 3 h before samples were taken and processed for 2D gel. Note the accumulation of SCJs in the *mms21∆C* strain

In recent years, many studies showed that the inactivation of the Smc5/6 complex and *sgs1∆* cells display similar phenotypes when exposed to DNA damage. They both accumulate recombination-dependent HJs upon replication stress (Ampatzidou et al. 2006; Bermudez-Lopez et al. 2010; Branzei et al. 2006; Liberi et al. 2005; Mankouri et al. 2009; Sollier et al. 2009), and show increased crossover frequencies between sister chromatids (de et al. 2006; Ira et al. 2003; Potts et al. 2006), indicating that their function is probably related. Interestingly, these defects are also seen when the SUMO ligase activity of the Smc5/6 complex is abrogated by the deletion of the C-terminal Siz/PIAS domain of Mms21/Nse2, a SUMO E3 ligase subunit of Smc5/6 [(Bermudez-Lopez et al. 2010) and Fig. 1].

Using a proteomic screen to look for substrates of Mms21, we identified several subunits within the Smc5/6 complex (Smc5, Smc6, Mms21, Nse3, and Nse4) and two subunits of the STR complex (Sgs1 and Top3) (Bermudez-Lopez et al. 2016). Although Sgs1 had been already described to be SUMOylated in response to DNA damage (Branzei et al. 2006; Lu et al. 2010), neither the SUMO ligase responsible for the modification nor the functional consequences were known. We and others recently demonstrated that the STR complex is able to recognise SUMOylated Smc5/6 complexes at sites of DNA damage (Bermudez-Lopez et al. 2016; Bonner et al. 2016). We proposed that Smc5/6 is attracted to DNA structures that require STR-dependent repair and that upon Smc5/6 engagement, an ATP-dependent remodelling of Smc5/6 drives the activation of the SUMO E3 ligase Mms21, previously demonstrated in (Bermudez-Lopez et al. 2015). As a consequence, SUMOylation of Smc5/6 subunits located at sites of DNA damage would ensue. Crucially, Sgs1 is able to specifically recognise SUMOylated Smc5/6 through two SUMO Interacting Motifs (SIMs) and through this be recruited to these damaged sites (Bermudez-Lopez et al. 2016). Once recruited, STR subunits are SUMOylated in the hands of Mms21, and this results in the activation of the recombinogenic function. Preventing either the recruitment of STR or its SUMOylation causes multiple recombination phenotypes, including decreased DNA end resection, accumulation of recombination-dependent structures at damaged forks and increases in crossover formation during DSB repair (Bermudez-Lopez et al. 2016). Taken together, a two-step role for Smc5/6 in recruiting and activating Sgs1 through SUMOylation seems to fine-tune STR complex functions during recombinational repair.

## Assembly of the STR complex in response to DNA damage

**Fig. 2:**
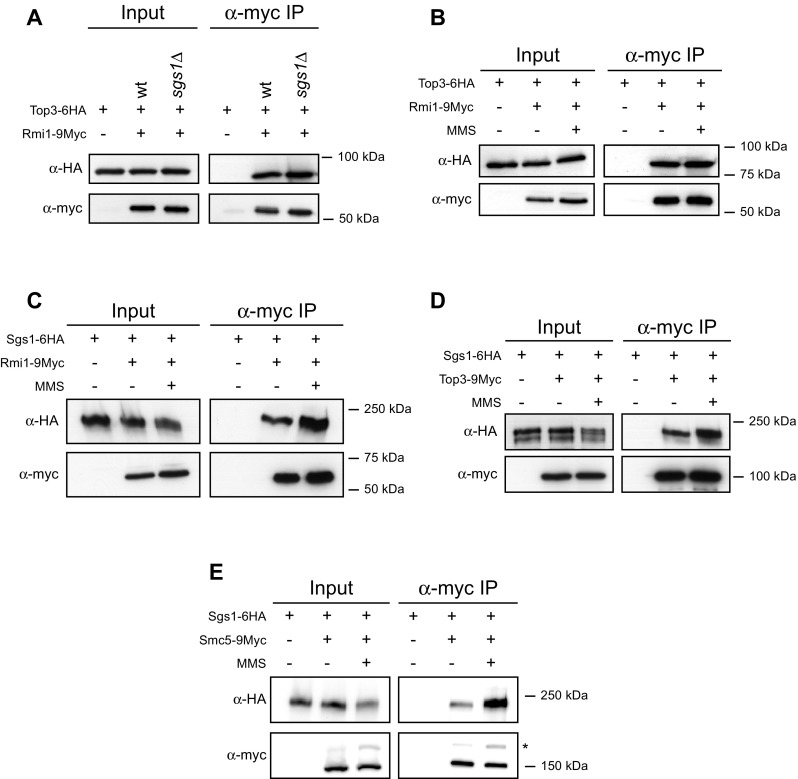
Assembly of STR complex in response to DNA damage. **a** Analysis of the Top3-Rmi1 interaction in wild-type and *sgs1∆* cells. **b** Analysis of the Top3-Rmi1 interaction. **c** Analysis of the Sgs1-Rmi1 interaction. **d** Analysis of the Top3-Sgs1 interaction. **e** Analysis of the Sgs1-Smc5 interaction. In **b**–**e**, the culture was split into two and one half was treated with 0.033 % MMS for 2 h before collecting them. Rmi1-9myc (**a**–**c**), Top3-9myc (**d**), or Smc5-9myc (**e**) was immunoprecipitated, and the co-immunoprecipitation of Top3-6HA (**a**, **b**) or Sgs1-6HA (**c**–**e**) was analysed by *Western blot*. An *asterisk* indicates SUMOylated Smc5-9myc

Although the requirement of all three subunits of the STR complex to form a fully functional complex has extensively been described, little is known about how the complex is assembled, particularly in response to DNA damage. During our analysis of STR regulation by Smc5/6, we investigated its effect on STR assembly. Our results revealed that the assembly of STR occurs in two distinct steps that are independent of Smc5/6 function. To analyse STR assembly, we generated strains expressing tagged copies of different STR subunits. First, we investigated the interaction between Top3 and Rmi1 tagging the two proteins (Top3-6HA and Rmi1-9myc). Pull-downs of Rmi1 resulted in a strong co-immunoprecipitation of Top3-6HA (Fig. 2a). This interaction did not require the presence of Sgs1, since both proteins co-immunoprecipitated with the same efficiency in *sgs1* null cells (Fig. 2a). This result is consistent with previous work, where Top3 and Rmi1 were shown to form a stable complex even in the absence of Sgs1 (Mullen et al. 2005). Interestingly, this interaction is not affected when cells are subjected to 0.033 % methyl methanesulfonate (MMS) for 2 h, which indicates that the Top3-Rmi1 interaction is independent of DNA damage (Fig. 2b). Next, we decided to explore the Sgs1-Rmi1 and Sgs1-Top3 interactions. In the unchallenged conditions, Sgs1, Top3, and Rmi1 form a heteromeric complex, in which the binding of Rmi1-Top3 subcomplex to Sgs1 is co-dependent (Mullen et al. 2005). Interestingly, both the interaction between Sgs1 and Rmi1 as well as between Sgs1 and Top3 increases when cells are exposed to DNA damage (Fig. 2c, d). Therefore, it is likely that in the unchallenged condition, two types of complexes exist inside cells, one formed by Sgs1-Top3-Rmi1 and another formed by Top3-Rmi1, and that the relative amount of Sgs1-Top3-Rmi1 complex, which likely represents functional STR, is increased upon DNA damage.

**Fig. 3:**
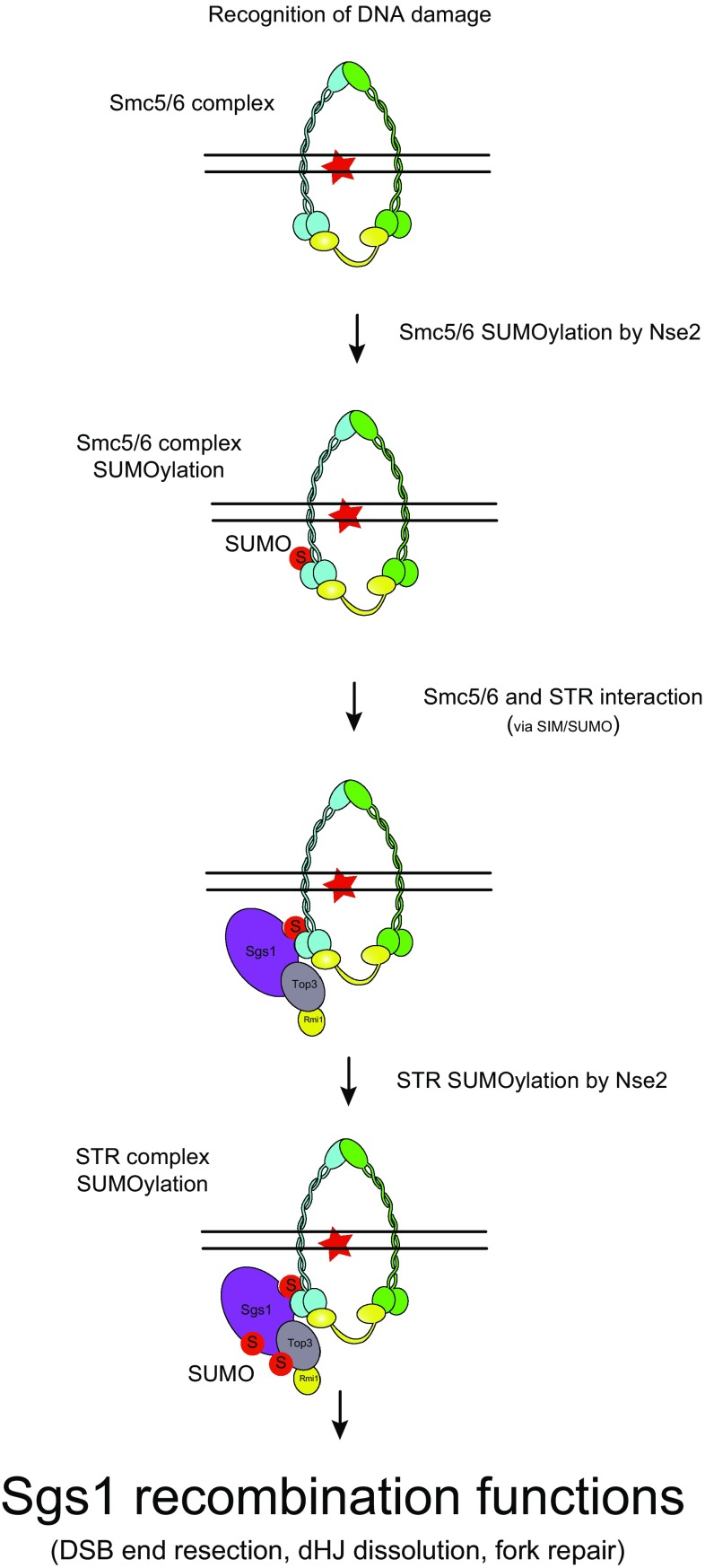
SUMOylation-dependent recruitment and activation of STR by Smc5/6 complex. Schematic representation of Sgs1’s pro-recombinogenic functions. Upon DNA damage that requires STR function, we propose that Smc5/6 is able to recognise suitable DNA substrates for Sgs1. Binding of Smc5/6 to DNA leads the activation of the SUMO E3 ligase Mms21 (Nse2). Nse2 then SUMOylates several subunits of the Smc5/6 complex. Sgs1 (as part of the STR complex) recruitment to these sites occurs via the recognition of SUMOylated Smc5/6 through the SIM domains of Sgs1. Then, Nse2 can SUMOylate Sgs1 and Top3. Nse2-dependent SUMOylation of Sgs1 and Top3 results in the activation of STR functions in recombination

Based on the fact that under DNA damage conditions, we observed a significant increase in the interaction between Sgs1 and the Smc5/6 complex (Fig. 2e), which occurs in a SUMO-SIM-dependent manner (Bermudez-Lopez et al. 2016), it is tempting to speculate a model, where Smc5/6 recruited to damaged DNA undergoes site-specific SUMOylation to trigger Sgs1 recruitment through Sgs1’s SIMs (Fig. 3). Once recruited STR becomes SUMOylated by the Smc5/6 subunit Mms21. This model provides an example of sequential site-specific SUMOylation (first Smc5/6 and then STR) that causes recruitment and activation of complexes involved in DNA damage repair.

## Post-translational modifications of Sgs1

BLM/Sgs1 has many roles in the repair of DSBs and replication forks (Larsen and Hickson 2013). These roles include both pro- and anti-recombinogenic activities; therefore, its activity must necessarily be tightly controlled to prevent detrimental effects to cells. In the past years, many studies have revealed that post-translational modification of BLM helicases can regulate some of these activities, particularly allowing fine-tuning of its functions. In fact, BLM phosphorylation, ubiquitination, and SUMOylation have been shown to control subcellular localization, interactions, and stability of this helicase [reviewed in (Bohm and Bernstein 2014)]. One of the earliest post-translational modifications described for BLM/Sgs1 was phosphorylation (Davies et al. 2004). The central checkpoint kinase ATM was shown to phosphorylate BLM in response to replication stress. Mutation of BLM phosphor-sites prevented recovery from replication arrests induced by hydroxyurea (HU) but did not affect SCE levels. This led to the proposition that BLM phosphorylation is particularly important for replication stress. Similar to BLM, Sgs1 is phosphorylated upon the activation of the intra S-phase checkpoint by Mec1 (ATR). Phosphorylated Sgs1 contributes synergistically to the further activation of Rad53 (the budding yeast CHK2 homolog), since the N-terminal region of Sgs1 interacts with Rpa70 and recruits Rad53 to stalled forks (Hegnauer et al. 2012).

As described earlier, Sgs1 is SUMOylated and this modification affects many Sgs1 functions (Lu et al. 2010; Bermudez-Lopez et al. 2016; Bonner et al. 2016). Sgs1 contains three lysine residues that lie within SUMO consensus sites (ΨKxE) at amino-acid positions 175, 621, and 831. Lys621 is the main acceptor site (Lu et al. 2010), but modification also occurs at Lys175 and Lys831. Sgs1 SUMO-deficient cells (*sgs1*-*3KR*) show an accumulation of SCJs when exposed to MMS and show a higher proportion of crossovers and an impaired DNA end resection upon DSBs (Bermudez-Lopez et al. 2016; Bonner et al. 2016). Like Sgs1, BLM is SUMOylated at different positions (Lys317 and Lys331), and SUMO-defective BLM cells also show high levels of SCEs (Eladad et al. 2005). Previous studies revealed that BLM/Sgs1 modification by SUMO leads to changes in the localization of the helicase in response to different DNA damage, but the mechanisms behind the altered localization remain unknown (Eladad et al. 2005; Ouyang et al. 2009). BLM is recruited to PML bodies through SIMs (SUMO interacting motifs), however, the SUMOylated target protein that BLM recognises is unknown (Zhu et al. 2008). In ALT cells, PML bodies contain telomeric DNA, and these types of PML bodies are known as APBs. Interestingly, APBs contain Smc5/6- and Smc5/6-dependent SUMOylation of the shelterin complex which is important to recruit telomeres to PMLs to form APBs (Potts and Yu 2007). It is tempting to speculate that Smc5/6 might promote telomeric recombination in ALT cells by coordinating recruitment of telomeres and BLM to APBs and by activating the pro-recombinogenic role of BLM in an analogous manner to what we have demonstrated for Sgs1 (Bermudez-Lopez et al. 2016; Bonner et al. 2016). Furthermore, *sgs1*-*K621R* mutant cells are defective in the production of type II survivors in the absence of telomerase (Lu et al. 2010), a yeast mechanism that is thought to be similar to the mammalian ALT pathway for telomere maintenance (Teng and Zakian 1999).Cases of cross talk between SUMOylation and phosphorylation have been demonstrated, for instance, SUMO-regulated phosphorylation has been linked to cell cycle control. However, the relationship between SUMOylation and phosphorylation in the function of Bloom helicases is currently unclear. We predict that interdependence of these two forms of modification is likely to occur, because some Sgs1 functions, particularly in replication forks, are affected by both modifications; nevertheless, future work should address how SUMOylation and phosphorylation fine-tune BLMs function, in which contexts they evoke opposing or similar functional outcomes in the roles played by these helicases.

## Conclusions/summary

Bloom helicases play key roles relevant to human health; therefore, understanding the molecular mechanisms of Sgs1/BLM function in different contexts is an important challenge. Bloom SUMOylation and its regulation by the Smc5/6 complex provide a new avenue for future studies. Presently, it is clear that Sgs1 is recruited through the recognition of SUMOylated Smc5/6 via SIMs; however, how Sgs1 SUMOylation affects its function remains unknown, one possibility is that SUMOylation modulates STR interactions with other proteins/complexes or DNA substrates; alternatively, this modification might affect helicase activity or protein stability. These are outstanding questions for the future, to gain a comprehensive understanding of STR and its relevance in human health and disease.

## Materials and methods

### Yeast strains

A list of strains used in this study is in supplementary Table 1. Epitope tagging and deletions were performed as described in (Goldstein and McCusker 1999; Janke et al. 2004)

### Yeast growth conditions

Yeast cells were grown in yeast extract peptone media (YEP) supplemented with glucose at 2 % final concentration. To induce DNA damage, cells were treated with 0.033 % MMS for 2 h before collecting them.

### Cell cycle synchronizations

To synchronize cells in G1, exponentially growing cultures were treated with α factor (Insight Biotechnologies) at a final concentration of 10^−8^ M until >95 % had been arrested in G1. The release from G1 was conducted by washing cells twice with pre-warmed medium and resuspending them in medium containing 0.1 mg/ml pronase from *Streptomyces griceus* (sigma, pronase).

### Co-immunoprecipitation

Co-immunoprecipitation analyses were performed as previously described in (Bermudez-Lopez et al. 2016).

### 2D Gel electrophoresis

2D Gel electrophoresis analyses were performed as previously described in (Bermudez-Lopez et al. 2016).

## Electronic supplementary material

Below is the link to the electronic supplementary material.

Supplementary material 1 (XLSX 25 kb)
